# Adapting to change: Clalit's response to the COVID-19 pandemic

**DOI:** 10.1186/s13584-021-00498-2

**Published:** 2021-11-30

**Authors:** Gil Lavie, Orly Weinstein, Yoram Segal, Ehud Davidson

**Affiliations:** 1 Clalit Health Services Management, Tel-Aviv, Israel; 2 Ruth and Bruce Rappaport Faculty of Medicine, Technion, Israel Institute of Technology, Haifa, Israel; 3 Faculty of Health Sciences, Ben-Gurion University of the Negev, Beer-Sheva, Israel

**Keywords:** COVID-19, VUCA, New normal, Organizational strategy, Proactive, Preventive, Virtual, Home-based

## Abstract

The COVID-19 pandemic is the most significant global health event of the past century. The profound and unexpected changes that it brought about have forced healthcare organizations to make far-reaching adjustments to accommodate the new reality. With the outbreak of the pandemic in Israel and the understanding of its consequences, Clalit Health Services (Clalit), the largest healthcare organization in Israel, rapidly mobilized in order to provide the best response possible from the perspective of both its patients and its employees. In the short term, four designated workgroups were established just days into the pandemic. Their task was to prepare operational work plans to achieve the following goals: providing the best possible treatment for COVID patients; maintaining the level of care for non-COVID patients; protecting healthcare personnel without compromising their competence and level of functioning; and beginning the process of post-crisis planning. In the context of the long term, and with the understanding that the changes in healthcare brought about by the COVID-19 pandemic would be long-lasting and irreversible, and would act as a catalyst in Clalit’s preparations for the future, Clalit has carried out the called-for modifications in its organizational strategy. This was based on the need to shift service and treatment foci from the hospitals to the community and the patient’s home and his cellular device, by means of strengthening Clalit’s strategic abilities to become more proactive, more digital and more home-based. In this article, we present a survey of Clalit’s preparations for the new reality in the short and medium terms, as well as the leveraging of insights gained during the first wave of the pandemic, with goal of revising Clalit’s long-term strategic plan. We conclude and point out the organizational abilities required for optimal response to future large-scale emergencies: The ability to quickly identify the need for change, respond quickly while harnessing the various parts of the organization in order to provide an agile and adaptive response, and facilitate long-term planning activity in parallel to providing an operational response in the short and medium terms.

## Background

The COVID-19 pandemic is one of the most important and influential events of the last century—a worldwide crisis that began in December 2019 in China and spread quickly throughout the world. The resulting morbidity and mortality has challenged the capabilities of healthcare systems and has had major social and economic impact. In view of the lack of certainty that characterized the pandemic from its onset, a rapid response that could be adapted to changing circumstances was called for. Clalit Health Services (herein: Clalit), the largest healthcare provider in Israel, mobilized to provide an optimal response to the new challenges. In the short and medium terms, it quickly adapted in an environment of uncertainty to the “COVID routine”, whose eventual duration was unknown. In the long term, it prepared for the “day after” in parallel to providing an ongoing response, based on the understanding that a new reality is taking shape.

In this article, we will survey the ways in which Clalit adapted to the new reality during the COVID-19 pandemic in the short and long terms and has leveraged the insights it gained in order to revise its strategic plan.

## Main text

Clalit is the largest of four health funds in Israel, which collectively insure the entire population, as part of a system of national mandatory healthcare coverage. Clalit insures and provides medical services to approximately 52% of Israel's population (4.7 million). It maintains a community network of about 1600 clinics located throughout the country and also owns and operates a third of Israel's general hospital beds. According to the strategic plan it adopted at the end of 2018, Clalit's overarching goal is to lead in providing high-quality, accessible, innovative and humane medical service to every patient, at any time and in any place. The strategic plan emphasizes the centrality of the patient, by seeking to create a comprehensive envelope of care in the community and in the hospitals and by means of supplementary health insurance and Clalit’s subsidiaries. Particular importance is attributed to maintaining continuity during the transfer of care between the community and the hospitals.

In late February 2020, the first COVID-19 patient was diagnosed in Israel. With the spread of the infection, restrictions were imposed on commerce and on mobility in the public domain, which eventually led to a lockdown at the beginning of April 2020. During this period, the healthcare system mobilized to provide an optimal response for COVID patients, whose numbers were growing rapidly. The situation was characterized by a high level of uncertainty as to the eventual scope of the pandemic and its duration, thus making it difficult to make the necessary adjustments.

An uncertain and complex reality is referred to in the strategic management literature using the acronym VUCA—volatility, uncertainty, complexity and ambiguity [1]. The VUCA concept was in use prior to the COVID crisis; however, the pandemic led to its amplification—accelerated change, greater uncertainty and complexity and lack of clarity in decision making. The organizational leaders in such a world need a clear vision and a well-defined organizational compass, a deep understanding of their organization’s strengths and weaknesses, the courage to initiate processes and decision making, and finally, flexibility and adaptiveness to deal with a rapidly changing reality [2].

Clalit faced two main challenges with the onset of the pandemic, as did the rest of the healthcare system:

1. *In the short and medium terms*—how to optimally and rapidly respond to the new conditions and to do so in a structured manner.
2. *In the long run*—how to plan and prepare a longer-term response, while at the same time dealing with the more immediate challenges.

### A rapid response in the short and medium terms: adapting to the changing reality

At the onset of the pandemic, Clalit already possessed a strategic plan and well-defined workplans derived from it. It was clear, already at an early stage, that we are facing a major crisis on both the national and international levels. A significant proportion of Clalit’s workplans were found to be irrelevant in dealing with the crisis and were disregarded in the effort to provide an optimal response to the virus and its effects. It was clear that we are entering a new and challenging period and that we must initiate an effective and up-to-date pan-organizational response as quickly as possible [3]. Already in March 2020, organizational workgroups were created in order to provide a structured response.

#### Creation of the workgroups

The workgroups were instructed to prepare and define a detailed and structured organizational response to the three main challenges facing Clalit in the short and medium terms:

1. To provide an appropriate response for COVID patients in the community and in the hospitals.
2. To provide an appropriate response for non-COVID patients in the community and in the hospitals.
3. To provide a defined and well-structured response to meet caregivers' needs.

Each workgroup was assigned a defined goal (Table 1). They were instructed to formulate an organizational workplan with a horizon of several months.

**Table 1:** The Clalit workgroups

Workgroup	The workgroup’s goal	The relevant time period
1. Providing an appropriate response for COVID patients	Planning an appropriate medical response for COVID patients in the community and in the hospitals, while maintaining a response capability that increases in scope over time, in accordance with the national reference scenario	Short and medium terms
2. Providing an appropriate response for non-COVID patients	Planning an appropriate medical response for Clalit’s non-COVID patients, while reducing excess morbidity to whatever extent possible	
3. Providing a response for caregivers	Planning a pan-organizational response in order to preserve the professional level and functioning of caregivers over time, with focus on the optimal treatment of the individual, minimizing burnout and maintaining the trust of the organization’s staff	
4. Planning for the “day after”	Initiating long-term preparations in view of the new reality—updating and modifying Clalit's strategic directions	Creating a foundation for preparedness in the medium and long terms

A fourth workgroup was established in order to plan for the “day after”, already during the first wave (March–May 2020). Its task was to examine, for the medium and long terms, the need to modify Clalit’s strategic directions in the face of the changing reality.

The workgroups began their work at the end of March 2020. They were formally appointed by Clalit’s Director General who defined their objectives and expected output (Table 2) and were given three weeks to complete their task.

**Table 2:** The workgroup foci of activity

*Workgroup I—An appropriate response for COVID patients:* The focus of this workgroup’s activity was split between the hospitals and the community		
	Hospitals	Community
Areas of focus	The preparedness of the hospitals to provide an appropriate medical response to COVID patients, according to the scope of hospitalization expected in the national scenario	Planning of an appropriate medical response for mild COVID patients and the quarantined who would be cared for at home or in hotels, according to the national reference scenario and including: - Establishment of routines for tracking and continuous communication - Defining of remote/personal monitoring and communication - Planning of a structured response to non-COVID medical problems among the quarantined and among COVID patients
	*Hospital infrastructure***—**preparation of designated care stations; electricity, oxygen, monitoring and care infrastructure	
	*Manpower* **-** Defining a quantitative target for the number of staff required (based on the assumption that some of them would be under quarantine or would become infected with COVID): physicians and nurses; and identification of critical staff, such as intensive care nurses and respiration technicians - Setting of a target for training of hospital staff in ventilation and appropriate use of protective equipment	
	*Equipment***—**Defining optimal inventory levels for protection equipment, respirators, monitors, and ECMO machines (according to consumption estimates based on the national scenario)	
	*Definition of designated care paths* in the hospitals	
	*Adoption of professional care guidelines –* uniform care protocols for all of the Clalit centers	

*Workgroup II—An appropriate response for non-COVID patients*: The focus of the workgroup’s activity was split between hospitals, the community and organization-level capabilities			
	Hospitals	Community	Pan-organizational
Areas of focus	Preserving pan-organizational capabilities in order to provide an appropriate medical solution to non-COVID patients in the ERs/wards/operating rooms/intensive care:	Defining a structured, proactive and hybrid (virtual and in-person) response in primary and consultative care for defined groups: - Chronic patients - Elderly patients at home under quarantine - Homecare patients - Seriously ill patients Preparations will include the creation of "smart lists": Identifying defined beneficiary groups based on Clalit's big-data models, which will make it possible to proactively contact them for the purpose of conveying information and offering services (both physical and virtual) that are suited to their needs For each group of patients, the planning of a response in the following areas: - Medical needs, such as the system of medical monitoring and tracking, the distribution of pharmaceuticals to the patient’s home, etc - Social, mental and logistic needs; providing an organizational response to mental health needs; and proactive tracking of socially isolated patients	Planning of a pan-organizational response in order to minimize excess morbidity, including the combining of forces between the community and the hospital:
	*Defining designated “clean” treatment paths—*Processual and physical separation, planning of stages for the opening of COVID wards and the continued operation of the other wards in parallel		*Acceleration of discharge from hospitals into the community* including a clear definition of the transfer process, the reinforcement of the hospital-community liaison staff and expansion of homecare units
			*Expansion of spatial activity* (in the geographic region in which the hospital and the Clalit district are located) by means of defining work processes and treatment paths that connect between the hospital and the region it serves
	*Manpower***—**Defining the required levels of manpower; assignment and training of manpower in order to achieve an appropriate response for non-COVID patients		*National information campaigns* in order to counter the reluctance of the public in seeking emergency medical treatment
			*Defining designated indexes of quality* for COVID treatment (indexes of treatment continuity, frequency of initiated contact with homecare patients, etc.)
	*Hospital outreach in the community—*Shifting of physical and virtual services to outside the hospital and making them accessible to the general public for consultative medicine and urgent ambulatory treatment (such as virtual operation of the hospital clinics, outsourcing services into the community such as mobile eye injection units, etc.)		

*Workgroup III—Solutions for the medical staff:*		
	Minimization of the number of infected and quarantined staff members	The organizational solution to minimize burnout, to support the individual employee and to maintain employee trust in the organization
Areas of focus	- Defining clear and structured work processes in patient care - Appointment of an institutional COVID coordinator, who will be responsible for revising the Corona procedures in a healthcare institution, the familiarly of the staff with the revised procedures and instructions, and supervision of their implementation - Prioritization of COVID screening tests and protective equipment	- Formulating a plan for conveying information and intra-organizational messages to employees in a continuous and transparent manner - Help desk for employee use - Focus on the individual—an organizational solution for family and economic problems, childcare and problems involving other members of the family - Consideration of employment flexibility and remote employment models - Increasing the number of employees trained as caregivers by shifting manpower from other relevant institutions and professions - Shifting of employees not directly involved in patient care to social or logistic tasks

*Workgroup IV—Preparing for the “day after”:*	
In parallel to the provision of solutions in the short and medium terms and based on the understanding that we are witnessing deep-rooted changes, this workgroup considered how Clalit should prepare for a period of “COVID routine”, which was expected following the conclusion of the first wave of infection. The workgroup began by creating an initial mapping of the long-term implications of the COVID-19 pandemic in order to provide answers to the following questions:	
1. Which elements of Clalit’s internal and external environments can be expected to change significantly in the future?	
2. How will the national exit from the first wave of infection and the shift to a “COVID routine” affect the achievement of Clalit’s current strategic goals and what are the risks that Clalit will face with the shift to a “COVID routine”?	
The product of this workgroup’s activity was used by the Clalit management to revise long-term strategic directions, as described in Sect. 2	

Each of the workgroups was instructed to adhere to the following guidelines:

- To carry out the work in a timely manner and in collaboration with representatives of the relevant managerial divisions in the organization, with emphasis on bringing together managers and representatives of the healthcare institutions.
- To produce a workplan based on measurable targets, a definition of who is responsible for each task and timetables for execution.
- Since the workgroups would be working in parallel to the ongoing response to the COVID-19 pandemic and the intensive efforts that involved, they did not include the most senior managers. Instead, their representatives or alternatively senior managers who had retired in the months prior to the pandemic were assigned to the workgroups. In this way, the activity of the workgroups did not disrupt the day-to-day operations in response to the pandemic.

The four workgroups included about 80 managers from all of Clalit’s divisions, i.e. from both Clalit’s managerial echelons and its healthcare institutions. In mid-April 2020, the workgroups presented their recommended workplans for Clalit’s response to the COVID-19 pandemic to the Clalit Executive and Board of Directors.

The output of the four workgroups was compiled into a single document and distributed to the Clalit management. It was then translated into Clalit’s revised workplans, which provided guidance for its activity in subsequent months. In this way, Clalit’s workplans were revised within a few weeks from the onset of the pandemic.

### Long-term planning and preparedness: revising Clalit’s strategy

“Never let a good crisis go to waste" – Winston Churchill, 1940

Although Clalit’s strategic plan was adopted in late 2018, the Clalit management initiated a process to revise it already during the first COVID-19 wave (March–May 2020). The goal was to improve Clalit’s long-term preparedness regardless of whether there would be additional COVID waves. The need for a revision arose in two contexts:

1. *The post-COVID New Normal***—**The COVID-19 pandemic led to fundamental and irreversible changes in modes of operation in a variety of systems, including the way in which health services will be provided in the future. Therefore, Clalit, like the rest of the healthcare system, invested its efforts in leveraging the progress made during the first wave in order to provide a suitable response to the new reality.
2. *Future challenges identified pre-COVID 19***—**The changes in Clalit’s strategy will improve its preparedness for the challenges of the future, as identified even prior to the pandemic, regardless of whether there are additional waves of COVID.

Following a structured organizational process of learning and drawing of conclusions, the revision of the Clalit strategic plan was carried out.

#### The new normal

"COVID-19 has changed health care, and some aspects of care delivery should not go back to the way they were" [4]

A "new normal" is a state to which an economy, society, etc. settles following a crisis, which will differ from the situation that prevailed prior to the crisis. The term has been employed in relation to major historical events, such as. World War I, the September 11 attacks, the financial crisis of 2007–2008, and the COVID-19 pandemic [5]. During the COVID-19 pandemic, the term has been increasingly used to refer to the changes in human behavior during or after the pandemic. This includes limiting person-to-person contact, maintaining social distancing, the adoption of practices and technologies that facilitate a modified routine of life, etc. [6].

Already during the first weeks of the pandemic, changes were rapidly implemented in order to deploy an appropriate medical response to the new conditions. At the onset of the pandemic, directives were issued that emphasized the importance of physical distancing and the avoidance of congregation. The restrictions on economic and social activity became increasingly stringent, up to the point of a stay-at-home-policy during the first lockdown (in April 2020). For a number of weeks, there was a decline of dozens of percent in the number of medical encounters in the community and in the hospitals due to the combination of two factors: a drop in supply (since hospitals had to curtail non-emergency activity in order to shift resources and be ready for dealing with a COVID surge) and a drop in demand (due to the fear among patients of becoming infected, which led to a decline of up to 50 percent in visits to medical facilities, including visits to the ERs for treatment of acute conditions). Within a few weeks, Clalit had to be prepared to provide a suitable medical response as part of a different supply model, one based on the following principles:

1. *A proactive approach*—Based on the ability to proactively identify and provide services to patients according to their specific characteristics and needs and in a way that is within the constraints imposed by the pandemic. The COVID-19 pandemic constituted an opportunity to develop and assimilate the use of artificial intelligence (AI) capabilities based on the big data possessed by Clalit, with the goal of identifying specific groups of patients and acting proactively for their benefit. A prime example is the ability to identify patients with increased risk of morbidity and mortality from COVID-19 using a risk predictor [7] based on algorithms and AI that make use of Clalit’s huge database. After identifying such a group, efforts were invested in outreach to its members and making the necessary preparations to provide them with care. This capability made it possible to send about 300,000 text messages to Clalit members who had been identified as having the highest risk levels already during the first COVID-19 wave. The text messages recommended remaining at home, provided guidance on how to behave in order to reduce the risk of infection and provided information on telemedicine and other remotely accessible medical services. Thus, the preventive- proactive measures that were based on advanced predictive tools made it possible to mitigate serious COVID illness in a way that was tailored specifically to the patient.
2. *Shift of physical services into the community, the patient’s home and the virtual domain*—Providing medical services in a location and in a manner that is suited to the patient. Already during the first weeks of the pandemic, Clalit began shifting resources from the hospitals to the community and the patient’s home, in a way that would provide an appropriate clinical response to the patient’s needs, avoid the need for the patient to physically come to the healthcare provider, reduce physical contact as much as possible and preserve the ability of the hospitals to serve seriously ill COVID patients. More than 90% of Clalit’s COVID patients during the first wave were taken care of in their homes, according to a structured care and monitoring protocol that included installation of temperature and saturation measuring instruments in the patient’s home, as well as routine contact and monitoring by healthcare providers in the community. There was a major ramp-up of homecare services, such as delivery of prescription drugs and visits by medical teams in order to carry out blood tests in the patient’s home. The shift into the community and into the patient’s home contributed to the wise use of the system’s resources, such that medical services were provided in a location and in a way that suited each patient and without overburdening the system. This included, for example, the hospitalization of a COVID patient only if his needs could not be suitably met in the community.
  In parallel to the physical shift into the community and the patient’s home, there was also a shift of services into the virtual domain. This involved the rapid assimilation of monitoring capabilities in patients’ homes and telemedicine encounters, mostly by means of telephone. During the first lockdown, about 50% of all primary medical encounters in the community were of this type and did not involve any physical contact between the caregiver and the patient. The telephone encounters made it possible to maintain contact between the patient and the mother-clinic, to carry out triage in order to channel patients to a physical examination if necessary and sometimes even to provide a full response to the patient’s needs. In the hospitals as well, COVID patients received care in a way that minimized physical contact and was based on online monitoring and communication to whatever extent possible. Technological capabilities were acquired and the medical staff gained extensive experience in using them; at the same time, they acquired the skills and abilities to provide care under these unique constraints.

The ability to assimilate preventive proactive data-based service and shift services into the virtual and home domains existed to some extent even prior to the COVID pandemic; however, the development of the system’s capabilities had progressed at a slow pace and on a small scale. The widespread clinical use of these capabilities encountered numerous barriers due to budget constraints, insufficient managerial focus and doubts among caregivers as to the feasibility of the services and the willingness of patients to adopt them. The COVID constraints and the involvement of all parts of the system in providing a suitable response to the emergency made it possible to achieve a quantum leap in a short period of time and therefore to redesign the model for service provision already during the pandemic. This essentially created a new reality of accessible services modified to meet the needs of patients, which reflected a transformation in service provision. The preservation and expansion of these capabilities will make it possible to continue providing high-quality, convenient and accessible medical service to patients even in the “new normal”, post-COVID era [4].

##### Mapping of the "New Normal"

**Fig. 1:**
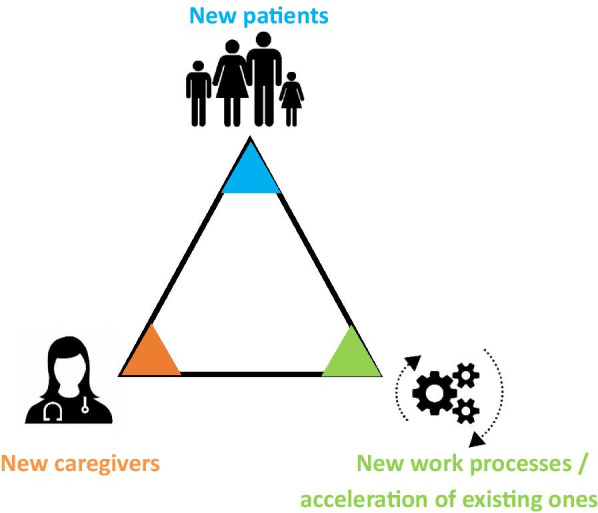
A mapping of the new reality

As part of the strategic mapping carried out by the Clalit executive, three main elements were identified which called for an all-encompassing adaptation to the new reality (see Fig. 1):

*"New" patients*

Clalit’s customers and patients received a new kind of service as part of the response to the COVID-19 pandemic: proactive medical service that is adapted to their needs and which provides them with a response even without having to physically visit the clinic, whether that involves virtual service or even physical service in their home. Clalit’s customers benefited from a configuration of service that provides a customized response to their needs and at a time and location they could choose. The "new" patients expect to continue and receive a similar standard of service and treatment, that should be leveraged and developed in the long term.

*"New" caregivers*

The Clalit staff have always been the organization’s source of strength. As mentioned above with respect to the activity of the third workgroup (Table 2—Workgroup III), efforts were made to maintain the staff’s ability to operate without interruption, to prevent burnout, and to provide optimal patient care. In addition to the care providers, who gained experience in caring for COVID patients in the community and in the hospitals while minimizing physical contact and making maximal use of video and online monitoring, other Clalit employees also had their first experience in the regular use of multi-participant videoconferencing (on ZOOM/Teams) and with the possibility of working from home by means of secure computer systems.

Continuing to integrate user-friendly virtual platforms that make wiser use of the care provider’s time and the implementation of flexible employment models will constitute an important part of Clalit’s strategic plan for the near future. These are important organizational capabilities that are suited to worker needs and which facilitate the optimal use of their time while encouraging a balance between work and family—their adoption is important whether or not there are additional waves of COVID.

*New work processes and the acceleration of existing ones*

As mentioned, already during the first wave of COVID we witnessed a significant shift of services from the hospitals to the community and the patient’s home. This shift, which was in accordance with the directives to minimize physical contact and was intended to maintain hospitalization capabilities for severe COVID patients, was based on three components, as mentioned above:

1. Proactive service.
2. Virtual service.
3. Home-based service

These were identified by us as strategic capabilities that should be the focus of Clalit’s long-term strategic plan, in the context of preparing for the new-normal post-COVID era.

#### Future challenges

**Fig. 2:**
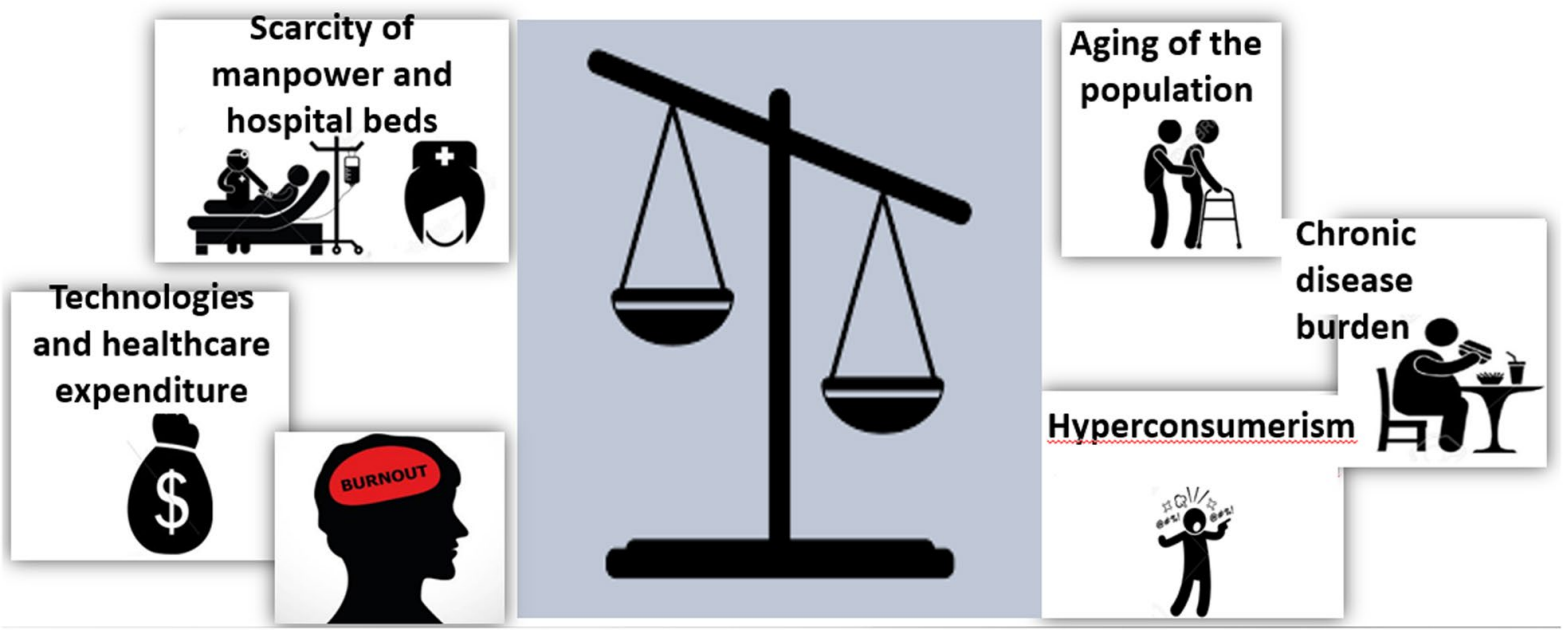
Supply–demand mismatch in the healthcare system

As part of the process to map future challenges and trends, which began already prior to the COVID pandemic, it was concluded that Clalit, like the rest of the healthcare system, faces the challenge of continuing to provide optimal healthcare to its patients, in view of the imbalance between forecasted demand and supply trends. This is a challenge that will become increasingly problematic as time passes (Fig. 2).

On the one hand—the heavy “weight” of demand is expected to increase due to a number of factors:

- *The forecasted aging of the population*—In 2040, the proportion of the 65 + age group within the population of Israel is expected to rise to 14.3 percent and that of the 85 + age group to 2.5 percent, in contrast to 11.1 percent and 1.4% respectively in 2015 [8]. These groups consume a particularly high level of services relative to their proportion of the population and the shift of system resources to meet their needs will affect the availability and quality of services provided to other groups.
- *An increase in chronic disease burden* even among younger patients—The diabesity epidemic [9], the increase in the consumption of fast food and the sedentary Western lifestyle will lead to increasing levels of chronic illness even among younger populations.
- *Modern consumption trends*—The healthcare consumer is increasingly demanding standards of service that are similar to those in other consumer domains, such as banking, culture and leisure. This means that service is continually available and accessible and sometimes meets no other need than to provide the consumption experience itself, a phenomenon known as hyperconsumerism [10]. The trend of Netflixication is a prime example, in which the consumption of service is initiated by the consumer, is tailored to the characteristics of the customer and proactively offers services that are fine-tuned to his previous consumption patterns.

On the other hand, there are a number of elements that will make it difficult to adjust supply to the increasing level of demand:

- *A national scarcity of medical and nursing manpower* and in hospital beds relative to other OECD countries [11].
- *The new technologies* that are being assimilated into the system are leading to spiraling levels of healthcare expenditure.
- *The burnout of care providers* which limits their ability to meet the growing burden on the system.

The aforementioned trends and the imbalance between expected demand and supply are expected to intensify in coming years and are liable to limit the availability and quality of services provided. The healthcare system must prepare for these trends in order to maintain the sustainability of the system, based on the understanding that duplicating the current medical service model is not feasible and will not provide a suitable response to the existing challenges. As part of the preparations to meet these challenges, the healthcare system and Clalit identified a need to implement systemic changes and to redesign healthcare provision, with the goal of achieving a wiser use of the system’s resources [12]. This includes both proactive prevention in order to lower the burden of chronic morbidity and the resulting “medical demand” on the one hand, and more efficient and prudent use of existing resources, according to the needs of the patient and without overburdening the system, on the other hand. This is to be accomplished by shifting the service foci to the appropriate care sites.

##### Preparations for future scenarios

The aging of the population and the increase in chronic illness will lead to a significant upward trend in the demand for medical services and a shortage in hospital beds. In order to respond to these needs, the hospitals will need to shift to a process of acutisation, which involves increasing the proportion of beds for complex or intensive care that can only be provided in a hospital setting. A large proportion of the hospitalization and ambulatory services do not have to be provided in a hospital setting and can be transferred to the community, where capabilities for providing service, diagnosis and treatment will be expanded. There will also be a shift from the treatment centers and community clinics into the patient’s home and onto his cellular phone. If a service can be provided at an appropriate level in the patient's home and will save the patient from having to come into the clinic, then the shift will have significant added value, both from the viewpoint of the patient, namely provision of service that is accessible and convenient and at a location and in a manner that suits his needs, and from the viewpoint of the system, namely wise use of its resources, such that medical services will be tailored to each individual patient and without burdening the system. This includes, for example, reducing unnecessary hospitalizations through disease prevention measures and treatment in the community, or providing an appropriate response in an outpatient setting in order to “preserve” expensive and scarce hospital beds for complicated and multidisciplinary patient care that cannot be provided outside a hospital setting. In the same way, a physical examination of a patient in a community clinic can be avoided if his needs can be met virtually or in his home, with the goal of utilizing the valuable time of the staff more efficiently in essential physical examinations.

As part of this approach, there is a need to expand acute capabilities of the hospital system to deal with a larger number of patients requiring hospitalization/intensive care/multidisciplinary staff capabilities. This is in parallel to the shifting of physical and virtual services from the hospitals to the community and the patient’s home, while strengthening preventive-proactive-personalized medical capabilities that will reduce chronic illness and expanding diagnostic and care capabilities in the community. This redeployment will enable Clalit to deal with the aforementioned supply–demand mismatch while providing high-quality and accessible service to its patients and using the system’s resources more wisely.

Already during the first weeks following the onset of the pandemic, the healthcare system prepared for extreme scenarios in which COVID patients would flood the hospitals and lead to a collapse of the system. In addition to the efforts to increase the capabilities of the hospitals to care for acute patients, the system made every effort to shift patients to outpatient settings. Within a few weeks from the onset of the pandemic, we were witness to processes that were identical to the long-term trends we had prepared for. The COVID pandemic therefore constituted an accelerated “general rehearsal” for the future. It brought to the fore and exacerbated challenges and shortfalls, and at the same time led to a rapid acceleration in processes and responses and to a revolution in the care provision model. Thus, the first wave of COVID served as a kind of “stress test” which made it possible to determine the relevance of Clalit’s response to the challenges of the future, and it constituted an opportunity to develop strategic capabilities of proactive, virtual and home-based medicine, which support the shift of services from the hospitals to the community, to the patient’s home and to his cellular phone. These capabilities will help Clalit and the healthcare system as a whole to provide a solution to long-term challenges, regardless of whether there are additional waves of COVID.

#### The learning process and the drawing of conclusions

**Fig. 3:**
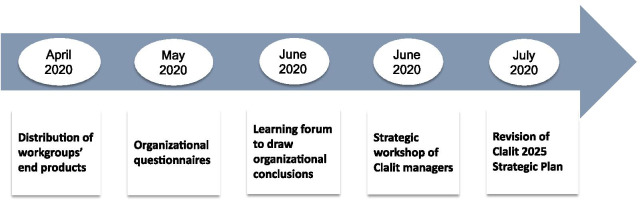
The learning process, the drawing of conclusions and the preparations for revising Clalit’s strategy

Following the conclusion of the first wave of the pandemic (May 2020), an organizational learning process was implemented in order to draw the appropriate organizational conclusions (Fig. 3). It was understood that the organization is facing a unique opportunity to learn from the COVID events and to leverage the insights gained.

##### Organizational questionnaires

As part of the process, questionnaires were distributed among all of the Clalit employees and managers. The goal of the questionnaire was to gain direct insight based on the experience of Clalit employees and managers in dealing with the first wave of infection and to formulate recommendations for improving Clalit’s preparedness for a period of “COVID routine” and possible future waves of infection.

##### Evaluation of the lessons learned from the COVID-19 pandemic

At the beginning of June 2020, the Clalit senior management and the managers of its healthcare institutions met to examine the lessons of the COVID-19 pandemic. The forum evaluated the conclusions to be drawn from Clalit’s response to the first wave of infection. Although the conclusions focused on the response to the COVID-19 pandemic, they also included insights that will be useful in improving Clalit’s future capabilities and performance in areas unrelated to the COVID-19 pandemic.

#### Revision of Clalit's strategy

In view of the lessons learned from the first wave of the pandemic and the leveraging of insights gained in order to strengthen Clalit’s long-term capabilities, a revision was carried out of the 2025 Strategic Plan. Clalit's overarching goal was revised and the “initiative” was added to it as a primary objective of the pan-organizational preparations, namely: to lead in providing high-quality, accessible, proactive, innovative and humane medical service to every patient, at any time and in any place.

At the center of Clalit’s strategic plan are three long-term objectives, which will have a deep-rooted effect on the future model of service provision: to become an organization that is more proactive and preventive, more digital and more home-oriented. These three pillars provide the foundation for a shift of care foci from the hospitals to the community, to the patient’s home and to his cellular phone, and they meet the needs of the “new” patients and care providers, while achieving wiser use of the system’s resources. Designated workgroups were established for advancing toward each of these objectives from a long-term strategic perspective:

1. *A proactive Clalit*—Maintaining a proactive approach to patients that combines medical and service values: it facilitates preventive and personalized medical measures, as well as supporting a high standard of patient experience in the offering of service that is tailored to his needs and desires, sometimes even before he realizes that he needs that service. This capability is based on the leveraging of Clalit’s big-data capabilities, the use of AI algorithms to predict clinical situations before they occur, identification of high-risk groups and the subsequent targeting of proactive and preventive efforts that are tailored to these patient groups. The data that is used in these efforts is both demographic and clinical, textual and numeric. It originates from both patient databases and the labs, from rapidly expanding genetic databases and from unstructured pathological and radiological data. The advancement of this proactive process requires the following:
  Development of abilities to gather and archive data from all information sources.
  Digitization of information to make it accessible for processing and advanced computerized analysis.
  Use of AI and machine learning capabilities which can be used to identify target groups among Clalit members.
  Development of the capability to contact targeted groups of patients.
  Monitoring the implementation of measures recommended by the patients.
  A number of pathbreaking predictive models have been assimilated in Clalit over the years as part of everyday medical practice (prediction and prevention of rehospitalization [13], prediction of osteoporotic fractures [14], etc.). The COVID pandemic has reinforced the need and the potential for expanding this powerful tool. This strategic workgroup is involved in encouraging the development and assimilation of various predictive models in the community and the hospitals, while transforming them into tools to be used routinely by care providers. This endeavor is supported by the development of advanced digital platforms with the goal of assimilating the predictive tools within routine medical practice and in order to achieve optimal communication with patients and involve them in this effort.
2. *A digital Clalit***—**Expanding the existing capacity for virtual medical encounters in a way that will provide a personalized response to the patients’ needs by means of an accessible, simple and convenient patient interface, as well as facilitate the wise use of the care providers’ valuable time. Virtual communication (by telephone or video) consists of a synchronic channel, namely an encounter that takes place simultaneously between the patient and the care provider and which provides greater access to medical service while avoiding the need for a patient to physically visit a medical center; and an asynchronic encounter, namely the ability of the patient to submit a request for service/information and the provision of a response at a later time (store-and-forward capability [15]). Asynchronic capabilities facilitate the integration of tools for information gathering and processing; the use of computerized tools in order carry out triage; and a precise response to the patient’s request, after the processing of the information he has submitted. In this way, an asynchronic response makes it possible to provide service that is suited to the patient and makes wiser use of the system’s resources and the care provider’s time, with human intervention occurring only after the cleansing of the information and its digitization.
  As in other countries [16], the rapid expansion in telemedicine during the first wave occurred due to the COVID restrictions that were imposed [17] rather than as a result of an evolutionary planning process [18]. In order to continue the efforts to adopt and expand this service, it will be necessary to structure the process according to the following metrics [19, 20]:
  Quality and safety: For which patient groups and clinical situations is the service most suited, such that it will meet standards of professionalism and quality, while reducing the potential risk of providing inappropriate service.
  The patient experience: Defining rules for the provision of appropriate medical service that supports patient-care provider communication and creates a desirable patient experience, even by virtual means.
  Risk management: Taking into consideration issues of privacy and data security and achieving an encounter that meets standards of risk management.
  Digital literacy and reduction of disparities: How to facilitate equal access and service that are tailored to various populations.
  The care providers: One of the main barriers to expanding virtual service prior to the COVID pandemic was the skeptical attitude among care providers with regard to its quality and the reluctance to make adjustments in order to provide it. The demonstration of its feasibility during the first wave was important but was not sufficient to convince medical staffs to continue using it nor to expand its usage. The medical staff should therefore be included in the planning process to achieve its optimal usage also in the future. There is a need to adapt digital platforms to the needs of the medical staff and to assimilate them within routine activity, such as to achieve wise use of the staff’s valuable time and not add to their workload or the difficulties they face. Furthermore, there is a need to adopt employment models and incentive mechanisms that will encourage staff members to use digital platforms, even outside of conventional working hours.
  Economic implications: Determining whether the virtual service provided in various clinical situations and to various groups of patients is in addition to in-person encounters, such that they only add to the system workload rather than creating real medical value.
3. *A home-based Clalit*—This workgroup examined the array of physical services that can be shifted to the community and the patient’s home from the traditional care centers in the hospitals and in the community.
  In the case of the hospitals, they examine services and procedures for which hospitalization could be shortened; that could be provided in outpatient clinics or that could be fully shifted to a community care center. Homecare has been provided by Clalit for many years by designated units (“continuing care units”) which provide care to chronically ill, bedridden, respirated and palliative care patients. Clalit also initiated homecare of acutely ill patients as a substitute for hospitalization about a year prior to the onset of the COVID pandemic. The trend toward homecare, which began prior to the pandemic in Israel and other countries, makes it possible for the patient to receive care in natural and familiar surroundings, while reducing the iatrogenic complications and deconditioning which is liable to accompany hospitalization. This is in addition to the freeing up of hospital beds for other patients [21]. The expansion of these capabilities and the large number of patients who have benefited from homecare during the COVID pandemic have highlighted the many advantages of this model for both the patient and the system and has increased the need to structure and expand this activity [22]—starting with surveys, diagnosis and prevention in the community and the patient’s home, use of monitoring and therapeutic devices for chronic, acute and sub-acute treatment at home, delivery of various services to the patient’s home and ending with the completion of the circle between the virtual domain, homecare and the patient’s clinic. This is a medical and service vector that is quickly gaining momentum [23–25] and is supported by digital and logistic capabilities; it in turn supports the shift of medical service foci into the community and the patient’s home.

All three of the workgroups sought to define where Clalit needs to be in 2025. In parallel, each of them presented an annual workplan for the coming year, whose implementation will facilitate progress toward achieving the long-term goals that were defined.

The revised strategic plan was distributed among Clalit’s employees and managers and was translated into annual workplans that began to be implemented already toward the end of 2020.

Alongside the strategic plan, a structured organizational plan for implementation was prepared, with the goal of conveying the main points of the strategic plan to all Clalit employees and transforming it into an accepted organizational language. The goal of the implementation plan is to enable each of Clalit’s tens of thousands of employees to understand the main points of emphasis in the organizational strategy, to translate it into his own individual “workspace” and to understand how it is relevant to him.

Within less than 6 months from the diagnosis of the first COVID patient at Clalit, Clalit’s 2025 strategic plan had been revised. The COVID events served as a catalyst for the updating of the plan, which will guide Clalit’s activity in the new reality in coming years [26].

## Conclusions and recommendations

During the COVID events, Clalit was called on to demonstrate managerial and organizational abilities that would enable it to cope with the pandemic in an optimal way in the short and long terms: the revision of the workplans that would provide an optimal response in the short and medium terms and an in-depth process to revise Clalit’s long-term strategic plan. These processes were based on the following key elements, which in our opinion, their implementation will assist healthcare organizations to respond optimally to future large-scale emergencies:

1. A rapid diagnosis of the changing trends and identification of the need to carry out modifications according to the various time horizons.
2. The ability to organize Clalit’s managerial and logistic capabilities in such a way as to facilitate long-term planning activity, in parallel to providing an operational response in the short and medium terms.
3. The ability to motivate and coordinate the various parts of the organization in order to provide an agile and adaptive response to current and future challenges.

The COVID-19 pandemic constituted a challenge for Clalit, as it did for the rest of the healthcare system and the State of Israel as a whole; however, it also constituted a rare and unique opportunity to carry out an in-depth examination of organizational insights, which led to an improvement in the organization’s preparedness for the challenges of the future.
